# A Rare Case of Single-Rooted Maxillary First Molar With Type II Canal Configuration

**DOI:** 10.7759/cureus.63076

**Published:** 2024-06-24

**Authors:** Hemal Bajaj, Rashmi Nair, Neetu Maurya, Aditya Patel, Saee Wazurkar

**Affiliations:** 1 Conservative Dentistry and Endodontics, Chhattisgarh Dental College and Research Institute, Rajnandgaon, IND; 2 Conservative Dentistry and Endodontics, Sharad Pawar Dental College and Hospital, Datta Meghe Institute of Higher Education and Research, Wardha, IND

**Keywords:** maxillary molar, endodontics, dental operating microscope (dom), cone-beam computed tomography (cbct), single rooted maxillary 1st molar, root canal treatment

## Abstract

The morphological variations in roots and root canals vary greatly in multi-rooted teeth making it a challenge for accurate diagnosis and effective endodontic therapy. In addition to using technology appropriately, this article highlights how important it is to have a complete understanding of root canal morphology. With the assistance of cone-beam computed tomography (CBCT) images and a dental operating microscope (DOM), successful endodontic treatment was performed on a single-rooted maxillary first molar with Vertucci’s type II canal configuration. CBCT and DOM proved to be valuable tools for the effective diagnosis and management of this atypical morphology.

## Introduction

Endodontic therapy requires the removal of all viable microbes and their toxins from the root canal. The intricate morphology of the root canal complicates treatment. Therefore, it is essential to have a thorough understanding of the many variations in the canal's anatomy. Failing to identify and address these variations can lead to unsuccessful root canal treatment, due to insufficient cleaning and shaping, obturation, and the presence of missing canals [1].

The maxillary molars have a failure rate of 31.60% due to their intricate morphology [2]. The root canal in the upper first molar varies significantly [3]. It is uncommon to have a single root in a maxillary molar and it is even more uncommon when it happens in a permanent maxillary first molar.

This study demonstrates an effective endodontic treatment of the upper first molar with distinct root canal morphology, presenting a single root with Vertucci’s type II canal configuration. The dental operating microscope (DOM) and cone beam computed tomography (CBCT) were essential in accurately diagnosing and managing this exceptional case.

## Case presentation

**Figure 1:**
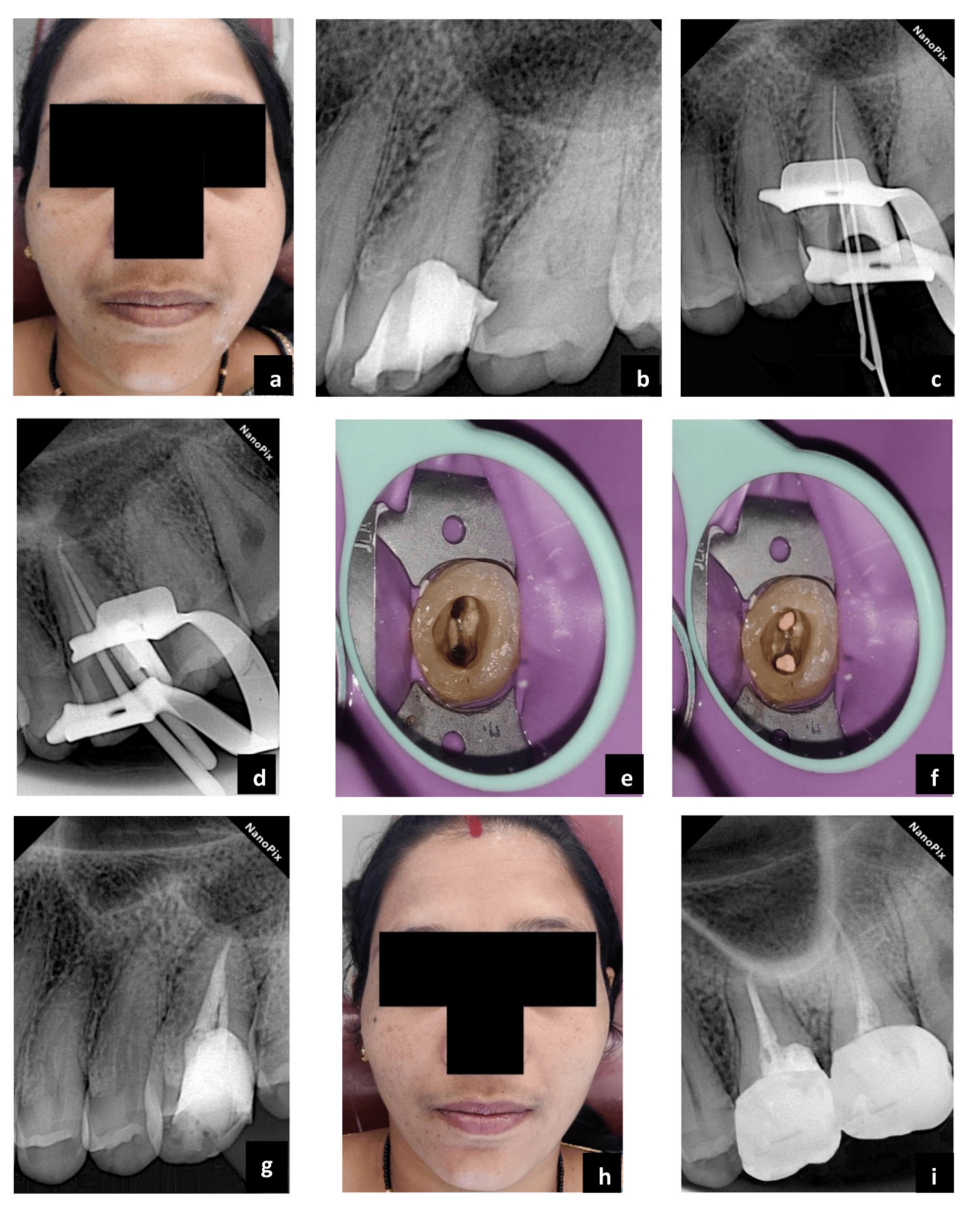
Endodontic treatment with #26 legend. (a) Preoperative extraoral photograph - facial asymmetry in the left cheek region, (b) preoperative radiograph of tooth #26, (c) working length radiograph, (d) master cone radiograph, (e) access opening clinical image, (f) postobturation clinical image, (g) postoperative radiograph, (h) postoperative extraoral photograph, and (i) 18 months follow-up image.

**Figure 2:**
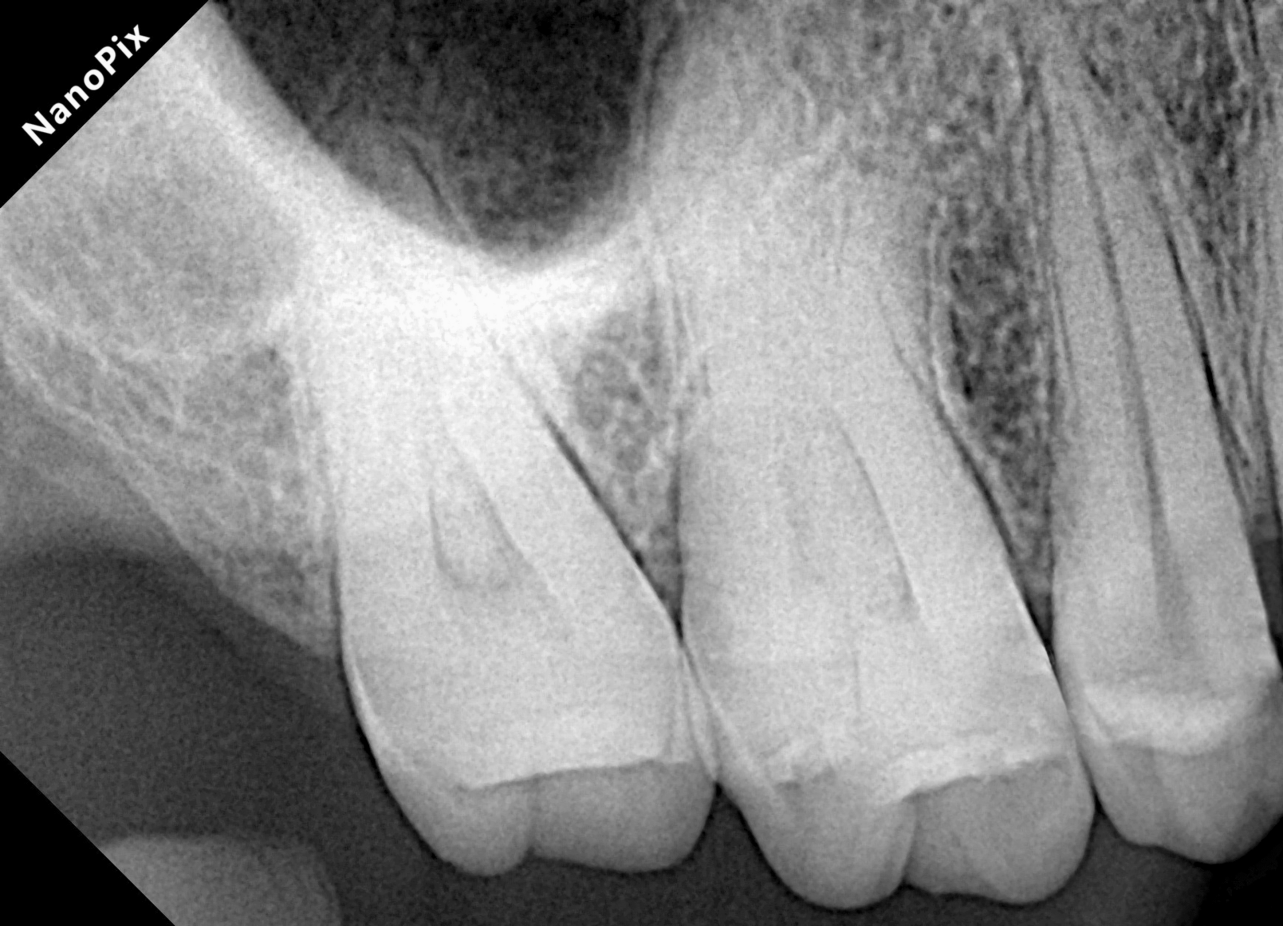
Intraoral periapical radiograph (IOPA) of tooth #16.

A 31-year-old female patient seeking urgent care reported at the Conservative Dentistry and Endodontics Department, Chhattisgarh Dental College and Research Institute, Rajnandgaon. She complained of pain and swelling in her left upper back region of the jaw for the past two days. The patient provided no contributing medical history. A private practitioner started endodontic treatment for tooth #26 four months back. In the left cheek region, facial asymmetry was observed (Figure 1, panel a). During the intraoral examination, tooth #26 responded positively to percussion and periodontal probing depth was normal. The cold test was done using Endo-Frost cold spray (Roeko; Langenau, Germany: Coltène/Whaledent Inc.) on the maxillary left first molar, along with healthy control teeth (maxillary right first molar, mandibular left second premolar, and mandibular left first molar). The control teeth exhibited a typical response of healthy pulp: a sharp brief pain that quickly dissipated once the stimulus was removed, suggestive of normal pulp vitality. Conversely, the maxillary left first molar did not respond to the cold stimulus. The electric pulp test was also performed, the upper left first molar showed no response compared to the adjacent and contralateral tooth. A large periapical radiolucency was observed during an intraoral periapical radiograph (IOPA) (Figure 1, panel b). The radicular pattern gave suspicion of the presence of one root only. The radiographic and clinical presentation led to a diagnosis of previously initiated therapy with acute apical abscess. It was also observed that the upper left first molar was asymmetrical compared to the upper right first molar, which exhibited three-root morphology (Figure 2).

Before initiating endodontic treatment informed consent was provided by the patient for the procedure. The tooth was then isolated using a rubber dam (Coltene Dental Dam Kit; Altstätten, Switzerland: Coltene), and both interim restoration and the carious lesion were removed under a dental operating microscope (Los Angeles, CA: Labomed, Inc.). Rebuilding of the missing distal wall of the tooth was done using Tetric N-Ceram (Schaan, Liechtenstein: Ivoclar Vivadent AG), access cavity preparation was modified and deroofing was done using round bur and safe end bur (BR-41, EX-24; Tochigi, Japan: Mani Inc.). The shape of the access cavity was ovoid rather than triangular or rhomboidal. The endodontic map indicated the presence of two orifices - buccal and palatal (Figure 1, panel e). The canals were initially negotiated using pre-curved K files #8 and #10 (Tochigi, Japan: Mani Inc.). During canal negotiation, a sudden canal blockage was noticed indicating a ledge in the palatal canal's apical third area, which was bypassed by pre-curving K files #6, #8, and #10. The determination of the working length was accomplished utilizing the Root ZX II apex locator (Kyoto, Japan: Morita Corporation), and its accuracy was verified through a periapical radiograph (Figure 1, panel c). Before initiating the root canal preparation, a rotary glide path was ensured for both canals by rotary pathfiles, 12-3%, 15-3%, and 19-3% (NineTen, India: NT Magic Pathfile). After preparation of the glide path, the following NT Rainbow (Dehradun, India: NineTen) sequence was used: 20-4%, 25-4%, 25-6%. The speed of the files was set at 400 rpm and torque at 2 NCm. The root canals were irrigated with 5.25% sodium hypochlorite (NaOCl) solution (Zodenta; Jodhpur, India: Neelkanth Healthcare P. Ltd) between each file. The irrigation process alternated between 5.25% NaOCl and 17% ethylenediaminetetraacetic acid (EDTA) solution (Jammu & Kashmir, India: Prevest DenPro). First, irrigation of the canals was done with a 5.25% NaOCl for 60 seconds and activated using the EndoActivator system (Long Island City, NY: Dentsply Sirona), with a tip #25, 0.04 taper, set at 10,000 cycles per minute. Next, distilled water was used for irrigation followed by 17% EDTA, again using the EndoActivator for another 60 seconds. Ultimately the canals were rinsed with distilled water and then dried using paper points (Seoul, Korea: DiaDent). A provisional restoration (Avue Temp; Mumbai, India: Dental Avenue) was applied to seal the tooth for further investigation and CBCT scanning to confirm the anatomical variation.

**Figure 3:**
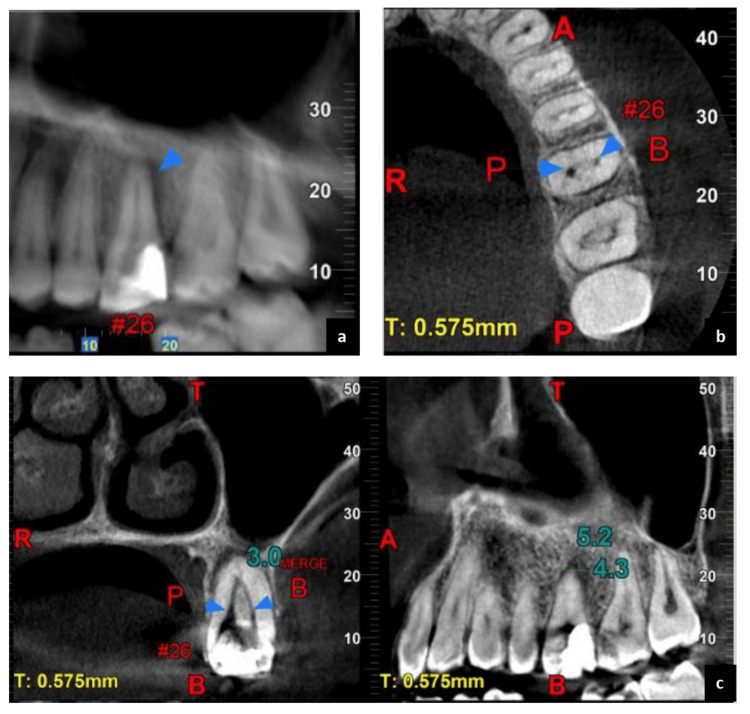
Cone beam computed tomography - (a) sagittal section, (b) axial section, and (c) coronal section.

The CBCT scan was analyzed in the coronal, axial, and sagittal planes. It confirmed the presence of an atypical morphological condition - single root with Vertucci’s type II canal configuration, as observed clinically and radiographically (Figure 3, panels a-c). At the second appointment, the extraoral swelling was completely healed and the provisional restoration was removed (Figure 1, panel h). Intracanal medicament (AvueCal; Mumbai, India: Dental Avenue) was used to fill the canals. Following this, the access cavity was sealed with provisional restoration (Avue Temp; Mumbai, India: Dental Avenue), and an appointment was scheduled after two weeks.

At the third visit, a rubber dam was applied, and provisional restoration was removed. The canals were irrigated following the same protocol as in the first visit. An appropriate size gutta-percha point (Seoul, Korea: DiaDent) was coated with a small amount of AH Plus sealer (Konstanz, Germany: DENTSPLY DeTrey GmbH) and compacted using the warm-vertical technique (Figure 1, panels d, f). A resin composite (Tetric N-Ceram; Schaan, Liechtenstein: Ivoclar Vivadent AG) was used to restore the access cavity (Figure 1, panel g) permanently. An 18-month follow-up radiograph confirmed the presence of bone regeneration, while a clinical examination demonstrated the absence of tenderness upon percussion (Figure 1, panel i).

## Discussion

Tooth anatomy is unpredictable, with numerous variations in both root formation and shape [4]. Various morphological variations in root canals of maxillary first molars have been documented over the years (Table 1). The variation in the number of roots was documented by Kottoor et al. and Gopikrishna et al. [4,5]. Extra roots were reported by Asghari et al. and Kottoor et al., while fused roots were documented by Chhabra et al. and Alzahrani et al. (Table 1) [6-9].

**Table 1:** Overview of previously documented variations in root canals of maxillary first molars. MB: mesiobuccal; DB: distobuccal; P: palatal; B: buccal

Type of study	Studies	Year	Number of roots	Number of canals	Country
Case report	Kottoor et al. [4]	2011	3	8 (3MB, 3DB, 2P)	India
Case report	Gopikrishna et al. [5]	2008	3 (1B, 2 P)	4 (2B, 2P)	India
Case report	Asghari et al. [6]	2015	4	4 (1MB, 1DB, 2P)	Iran
Case report	Kottoor et al. [7]	2011	4	4 (1MB, 1DB, 2P-C shaped)	India
Case report	Chhabra et al. [8]	2013	1	1	India
Case report	Alzahrani et al. [9]	2023	2	2 (1B, 1P)	Saudi Arabia

In more than 95.9% of maxillary first molars, three roots are found, whereas only 3.9% have two roots. Root fusion occurs in approximately 5.2% of cases. C-shaped canals and conical roots are rare, appearing in just 0.12% of cases [10]. A small percentage of the population (0.25% in Korea [11] and 0.9% in India) present with a single root in the upper first molar, with equal distributions of Vertucci’s type V and type I configurations (0.5%) [12].

This study explains endodontic therapy for the upper first molar with a single root and Vertucci's type II canal anatomy. The inorganic smear layer and organic debris were removed by irrigation with 17% EDTA and 5.25% NaOCl, respectively. The canals were sealed three-dimensionally using warm vertical compaction of gutta-percha [13]. Additionally, asymmetry was observed in the left and right quadrants. According to a study, 70% and 81% of roots and their canal showed symmetrical morphology between the left and right sides of the jaw [14].

In specific situations, conventional 2-dimensional X-rays may not offer sufficient diagnostic information for clinicians to understand the complex morphology of the canal completely. To address these challenges, CBCT imaging is employed, enabling a three-dimensional visualization of teeth and their surrounding structures [9]. Moreover, incorporating a dental operating microscope (DOM) into regular clinical practice can enhance the identification and management of canals, due to its significantly increased illumination and magnification [8,15]. In the present case, only one root and two canals (Vertucci type II) were suspected from multiple angled radiographs, confirmed with CBCT, and managed with root canal therapy.

## Conclusions

Unusual root morphologies pose a challenge for clinicians. It is essential to implement a meticulous treatment plan involving the thorough interpretation of preoperative radiographs from different angles and the application of advanced technologies such as CBCT and DOM. This is crucial for effectively managing aberrant anatomies in the root canal and enhancing the prognosis.
